# Gremlin induces cell proliferation and extra cellular matrix accumulation in mouse mesangial cells exposed to high glucose via the ERK1/2 pathway

**DOI:** 10.1186/1471-2369-14-33

**Published:** 2013-02-11

**Authors:** Haixia Huang, Haiying Huang, Ying Li, Maodong Liu, Yonghong Shi, Yanqing Chi, Tao Zhang

**Affiliations:** 1 Department of Nephropathy, The Third Hospital of Hebei Medical University, Shijiazhuang 050051, China; 2 Hebei Provincial Hospital of Armed Police, Shijiazhuang, 050081, China; 3 Bethune Military Medical College of PLA, Shijiazhuang, 050081, China; 4 Department of Pathology, Hebei Medical University, Shijiazhuang, 050017, China

**Keywords:** High glucose, Gremlin, ERK1/2, Cell proliferation, Transfection, Glomerular mesangial cells

## Abstract

**Background:**

Gremlin, a bone morphogenetic protein antagonist, plays an important role in the pathogenesis of diabetic nephropathy (DN). However, the specific molecular mechanism underlying Gremlin’s involvement in DN has not been fully elucidated. In the present study, we investigated the role of Gremlin on cell proliferation and accumulation of extracellular matrix (ECM) in mouse mesangial cells (MMCs), and explored the relationship between Gremlin and the ERK1/2 pathway.

**Methods:**

To determine expression of Gremlin in MMCs after high glucose (HG) exposure, Gremlin mRNA and protein expression were evaluated using real-time polymerase chain reaction and western blot analysis, respectively. To determine the role of Gremlin on cell proliferation and accumulation of ECM, western blot analysis was used to assess expression of pERK1/2, transforming growth factor-β1 (TGF-β1) and connective tissue growth factor (CTGF). Cell proliferation was examined by bromodeoxyuridine (BrdU) ELISA, and accumulation of collagen IV was measured using a radioimmunoassay. This enabled the relationship between Gremlin and ERK1/2 pathway activation to be investigated.

**Results:**

HG exposure induced expression of Gremlin, which peaked 12 h after HG exposure. HG exposure alone or transfection of normal-glucose (NG) exposed MMCs with Gremlin plasmid (NG + P) increased cell proliferation. Transfection with Gremlin plasmid into MMCs previously exposed to HG (HG + P) significantly increased this HG-induced phenomenon. HG and NG + P conditions up-regulated protein levels of TGF-β1, CTGF and collagen IV accumulation, while HG + P significantly increased levels of these further. Inhibition of Gremlin with Gremlin siRNA plasmid reversed the HG-induced phenomena. These data indicate that Gremlin can induce cell proliferation and accumulation of ECM in MMCs. HG also induced the activation of the ERK1/2 pathway, which peaked 24 h after HG exposure. HG and NG + P conditions induced overexpression of pERK1/2, whilst HG + P significantly induced levels further. Inhibition of Gremlin by Gremlin siRNA plasmid reversed the HG-induced phenomena. This indicates Gremlin can induce activation of the ERK1/2 pathway in MMCs.

**Conclusion:**

Culture of MMCs in the presence of HG up-regulates expression of Gremlin. Gremlin induces cell proliferation and accumulation of ECM in MMCs. and enhances activation of the ERK1/2 pathway.

## Background

Gremlin is a 184-amino acid protein, which is a member of the structural cysteine knot superfamily [1,2]. This protein is evolutionarily conserved and the human Gremlin gene has been mapped to chromosome 15q13-q15 [3,4]. Functionally, Gremlin belongs to a novel family of BMP antagonists that includes the head-inducing factor Cerberus and the tumor suppressor DAN [5]. Under basal conditions, Gremlin is present at relatively low levels in the adult kidney [6]. However, Gremlin is overexpressed in human mesangial cells exposed to high extracellular glucose *in vitro*[7]. TGF-β, when added to serum-restricted human mesangial cells, has been found to augment Gremlin expression, but the stimulatory effect of high glucose on Gremlin expression can be attenuated by the addition of anti-TGF-β antibody [7]. This evidence indicates that Gremlin expression is induced by TGF-β under diabetic conditions. These data suggest a pathogenetic role for Gremlin in DN.

Gremlin gene expression is modulated by hyperglycemic induction of the ERK1/2 in bovine retinal pericytes in response to elevated glucose and in the retina of the streptozotocin induced diabetic mouse [8]. ERK1/2 is a member of the MAPK family [9]. The ERK1/2 pathway becomes activated in mesangial cells cultured under high-glucose conditions. This pathway and regulates many transcription factors that control cell growth and ECM gene expression [10]. In this way, it appears that the activation of ERK pathway may be one of the major mechanisms involved in high glucose-induced glomerular injury.

However, the molecular mechanism of Gremlin’s involvement in DN has not been fully elucidated, especially the role of Gremlin on activation of the ERK1/2 pathway. For this reason, this study was designed to investigate the role of Gremlin on cell proliferation and accumulation of ECM in MMCs under HG conditions and to explore the relationship between Gremlin and the ERK1/2 pathway.

## Methods

### Cell line and reagents

MMCs were purchased from the American Type Culture Collection (Manassas, VA, U.S.). Antibodies against TGF-β1 was obtained from Santa Cruz Biotechnology (Santa Cruz, CA, U.S.). Antibodies against Gremlin and CTGF were obtained from Abcam Biotechnology (Abcam, CA, U.S.). Antiphosphotyrosine antibody for ERK1/2 and antibody for ERK1/2 were purchased from Cell Signaling Technology (Beverly, MA, U.S.). The RT-PCR systems were obtained from Takara Biotechnology (Dalian, China).

### Construction of expression plasmids

The negative control plasmid (pEGFP-N1) and Gremlin plasmid (pEGFP-N1-Grem1), were designed and produced by Shanghai Yingweixin Co. The primer sequences for amplification of Grem1 gene are as follows: Grem1-F: 5^′^-ATGAATCGC ACCGCATACACTG-3^′^, Grem1-R: 5^′^-ATCCAAGTCGATGGATATGCAA-3^′^. The negative control plasmid (pGenesil1.1) and Gremlin siRNA plasmid (pGenesil1.1-shGrem1) were designed and produced by Wuhan Jingsai Co. The pGenesil1.1-shGrem1 was designed to target the sequence 5^′^-GCACATCCGAAAGGAGGAA-3^′^. The short chain oligonucleotide was as follows: F: 5^′^-CACCGCACATCCGAAAGGAGGAAT TCAAGACGTTCCTCCTTTCGGATGTGCTTTTTTG-3^′^, R: 3^′^–AGCTCAAAAA AGCACATCCGAAAGGAGGAACGTCTTGAATTCCTCCTTTCGGATGTGC-5^′^, structure: BamHI + Sense + Loop + Antisense + Endsignal + SalI + HindIII. To determine the efficiency of the plasmids, MMCs cultured under NG were transfected with these plasmids. Gremlin expression was evaluated using western blot analysis and real-time polymerase chain reaction. The plasmids were all found to be effective and deemed suitable for the study.

### Cell culture and transient transfection

MMCs were grown in DMEM-F12 (Gibco BRL) containing 10% FBS, 100 U/ml penicillin, 100 mg/ml streptomycin, and 5% CO_2_:95% air at 37°C. Prior to use, cells at 80% confluence were synchronized by culturing in serum-free medium for 24 h. (1) To investigate the effects of glucose on the expression of Gremlin and pERK1/2, MMCs were stimulated with NG plus 24.5 mM mannitol (M) or HG for 0, 6, 12, 24 and 48 h. At the end of each time interval, total RNA and protein of the cells were extracted for Gremlin and pERK1/2 expression. All experiments were repeated three times. (2) In order to determine the direct effects of expression plasmids, the cells were transfected with 2.0 μg of plasmid with 4 μl of Lipofectamine 2000 (Invitrogen, CA, U.S.) in 2 ml of serum-free DMEM-F12 medium. At 6 h after transfection, the medium was replaced with normal DMEM-F12 medium containing 10% fetal bovine serum and cells were incubated for 24 h. Then the cells were cultured for 24 h in DMEM-F12 containing HG or NG. At 24 h after HG stimulation, total RNA and protein were extracted from the cells for analysis of Gremlin, TGF-β1, CTGF, and pERK1/2 expression and culture medium was collected for measurement of the concentration of collagen IV. All experiments were repeated three times.

### Real-time polymerase chain reaction (RT-PCR)

The cells were harvested and total RNA was extracted using TriZol Reagent (Introvigen, Carlsbad, CA, U.S.) according to the manufacturer’s instructions. RNA concentrations were determined using a Nanodrop ND-2000 spectrophotometer (Nanodrop Technologies, Wilmington, DE, U.S.), and the complementary DNA (cDNA) was synthesized from the total RNA (500 ng) using a PrimeScript® RT Reagent Kit (DRR037S, Takara Biotechnology, Dalian, China) in accordance with the manufacturer’s instructions. Then the cDNA was subjected to real-time PCR using a SYBR® Premix Ex TaqTM (DRR041A, Takara Biotechnology, Dalian, China). Each real-time PCR reaction consisted of 2 μl of diluted RT product, 10 μl SYBR® Premix Ex TaqTM and 200-nm specific primer pairs in a total volume of 20 μl. The reactions were performed on a M × 3005P real-time PCR System (Agilent Technologies) an initial 30 s incubation at 95°C followed by 40 cycles (95°C for 5 s, 60°C for 30 s 72°C for 20 s) after. The fold change in the mRNA levels of each gene was calculated using the ΔΔCt method. The housekeeping gene 18S rRNA served as an internal control. All PCR primers were synthesized by Generay Biotech, China. The primer sequences and the amplified lengths used in the MMC study were as follows: Gremlin (sense: 5^′^-AGCAAAAGGGTTTTCCTGAT-3^′^, antisense: 5^′^-AATGGTCAGCATTTCACCCT-3^′^, 200 bp), 18S rRNA (sense: 5^′^-ACA CGGACAGGATTGACAGA-3^′^, antisense: 5^′^-GGACATCTAAGGGCATCACAG- 3^′^, 238 bp).

### Western blotting

Total protein was extracted from cultured cells as described previously [11]. The protein extracts were separated using 10% SDS-PAGE and then transferred to PVDF membranes. The membranes were incubated overnight with rabbit polyclonal anti-Gremlin, ERK1/2, pERK1/2, CTGF, β-actin (1:1000), and TGF-β1 (1:200) antibodies at 4°C. Then the membranes were incubated with horseradish peroxidase-conjugated goat anti-rabbit IgG (1:5000) and then exposed to X-ray film using an enhanced chemiluminescence system (Pierce, IL, U.S.). The intensity of the bands was measured using LabWorks 4.5.

### BrdU–Enzyme-linked Immunosorbent Assay (ELISA)

MMC proliferation was determined by measuring BrdU incorporation using a BrdU incorporation assay (Roche Molecular Biochemicals, Mannheim, Germany) according to the manufacturer’s instructions. Briefly, 5,000 MMCs/well seeded in a 96-well plate were pulse-labeled for 2 h with 10 M BrdU. Cells were then incubated for 30 min with diluted, peroxidase-conjugated anti-BrdU antibody. Absorbance values were measured at 450 nm using an ELISA reader (Bio-Rad iMark, Richmond, CA, USA).

### Radioimmunoassay (RIA)

At the end of each time point, the supernatants from the cell cultures were collected to measure collagen IV content. The concentration of collagen IV was determined by radioimmunoassay (RIA) using commercially available kits (Beifang Inst. Biotechnology, Beijing, China) according to the manufacturer’s instructions.

### Statistical analysis

The data were normally distributed, as determined using a Kolmogorov-Smirnov test. Comparisons among groups were conducted using one-way ANOVA. When the F value was significant (*P* < 0.05), the Student–Newman–Keuls Test and least significant difference procedure were performed to evaluate differences between the means. All data were expressed as mean ± standard deviation (SD) and analyzed using SPSS 18.0 for Windows. Statistical significance was defined as *P* < 0.05.

## Results

### Effects of high glucose on the expression of Gremlin in MMCs

**Figure 1:**
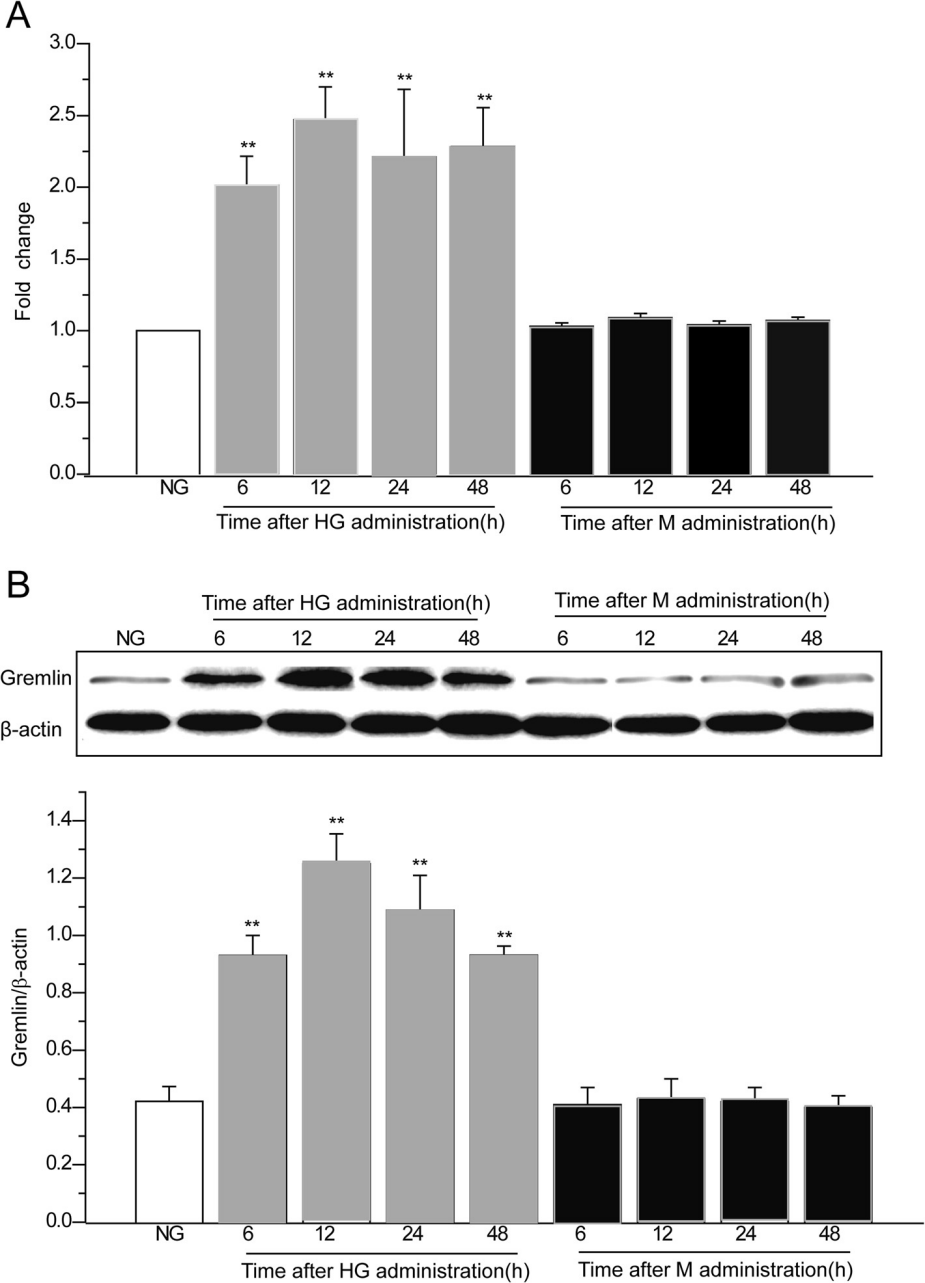
**Effects of HG on Gremlin protein and mRNA in MMCs over time.** MMCs were cultured in media containing 5.5 mM glucose (NG), then stimulated with NG plus 24.5 mM mannitol (M) or 30 mM glucose (HG) for 6, 12, 24, and 48 h. At the end of each time interval, total RNA and protein of the cells were extracted for Gremlin mRNA and protein expression. (**A**) The level of Gremlin mRNA expression was detected using real-time polymerase chain reaction (RT-PCR) analysis. (**B**) The expression of Gremlin protein was analyzed using western blot. Values are expressed as means ± S.E.M. *P < 0.05, **P < 0.01 vs. NG.

In order to confirm the effects of glucose on the expression of Gremlin, MMCs were cultured in media containing 5.5 mM glucose (NG), then stimulated with NG plus 24.5 mM mannitol (M) or 30 mM glucose (HG) for 6, 12, 24, and 48 h. The expression of Gremlin mRNA was detected using real-time polymerase chain reaction (RT-PCR) analysis. We found that the level of Gremlin mRNA increased within 6 h of HG stimulation (2.01 ± 0.20 fold, *P* < 0.01, vs NG) and peaked at 12 h (2.47 ± 0.23 fold, *P* < 0.01, vs NG) (Figure 1A). As shown in Figure 1B, increased expression of Gremlin protein was further confirmed by western blot. Consistent with the results observed at the mRNA level, western blot analysis also showed a significant increase in Gremlin protein expression under high glucose conditions (30 mM) at all time intervals. This increase peaked at 12 h (*P* < 0.01, vs NG). In addition, no differences were found in MMCs cultured under conditions of NG plus mannitol among different time points. These finding reveal that Gremlin was involved in HG-induced effects in MMCs.

### Transient transfection of MMCs with expression plasmids

**Figure 2:**
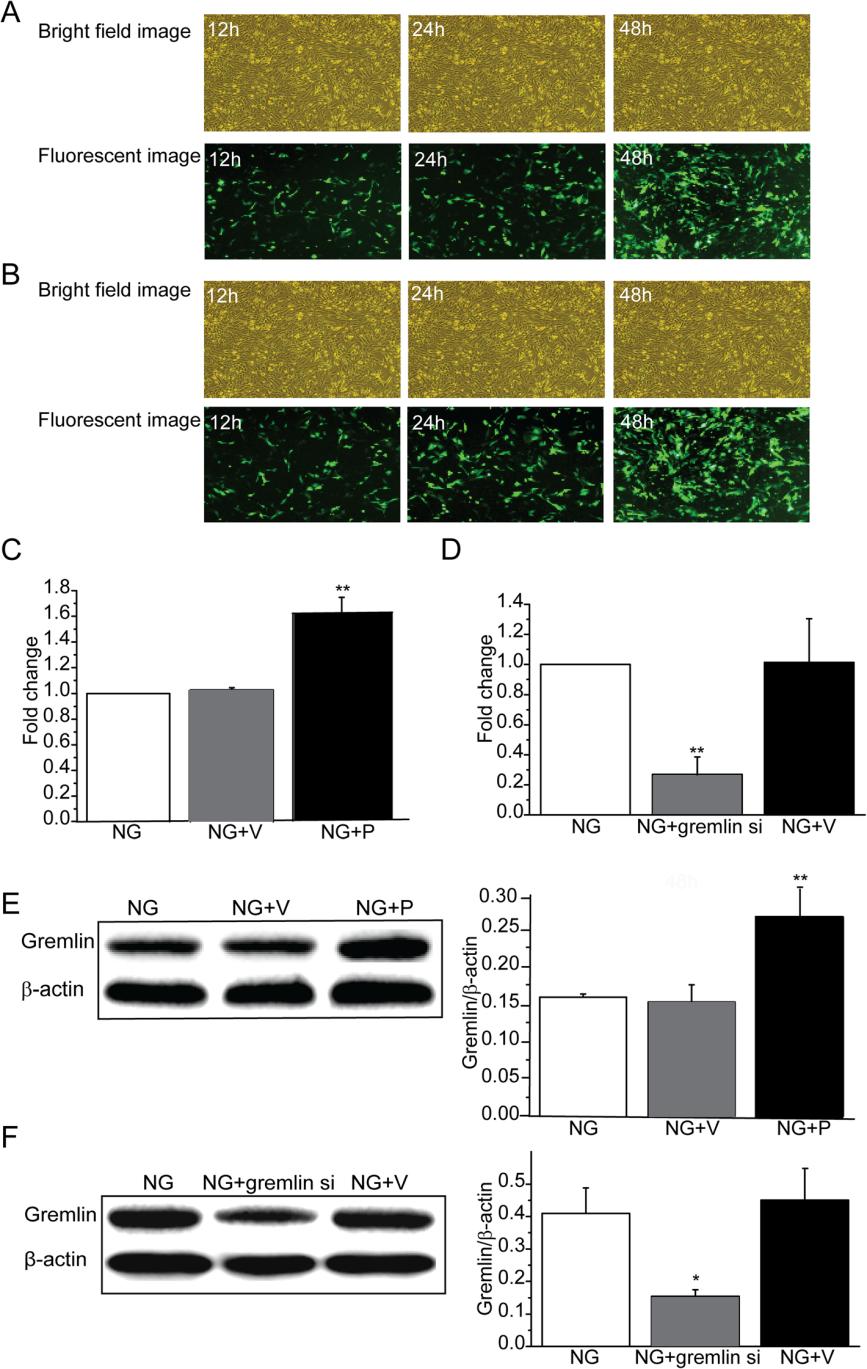
**Effects of expression plasmids on the expression of GFP and Gremlin in MMCs.** Transient transfection of MMCs with Gremlin plasmid (pEGFP-N1-Grem1) and Gremlin siRNA plasmid (pGenesil1.1-shGrem1) induced GFP expression, indicating successful transfection. GFP expression was observed at 12, 24, and 48 h after transfection. MMCs were transfected with Gremlin plasmid (pEGFP-N1-Grem1), Gremlin siRNA plasmid (pGenesil1.1-shGrem1), or their corresponding negative control plasmids (pEGFP-N1or pGenesil1.1). They were then incubated with NG (5.5 mM D-glucose). At 48 h after transfection, total RNA and protein were extracted from the cells for Gremlin mRNA and protein expression. (**A**) GFP expression was observed through the comparative analysis of bright-field image and corresponding fluorescent image at 12, 24, and 48 h after Gremlin plasmid transfection (200×). (**B**) GFP expression was observed through the comparative analysis of bright field images and corresponding fluorescent images at 12, 24, and 48 h after Gremlin siRNA plasmid transfection (200×). (**C**) The expression of Gremlin mRNA was detected by real-time polymerase chain reaction (RT-PCR) analysis at 48 h after Gremlin plasmid transfection. (**D**) The expression of Gremlin mRNA was detected using RT-PCR analysis 48 h after Gremlin siRNA plasmid transfection. (**E**) The expression of Gremlin protein was analyzed using western blot analysis 48 h after Gremlin plasmid transfection. (**F**) The expression of Gremlin protein was analyzed using western blot analysis 48 h after Gremlin siRNA plasmid transfection. NG: MMCs incubated with 5.5 mM D-glucose; NG + P: MMCs transfected with pEGFP-N1-Grem1 plasmid and incubated with 5.5 mM D-glucose; NG + gremlin si: MMCs transfected with pGenesil1.1-shGrem1 plasmid and incubated with 5.5 mM D-glucose; NG + V: MMCs transfected with pEGFP-N1 or pGenesil1.1 plasmid and incubated with 5.5 mM D-glucose. Values are expressed as means ± S.E.M. *P < 0.05, **P < 0.01 vs. NG.

Transient transfection of MMCs with Gremlin plasmid (pEGFP-N1-Grem1) or Gremlin siRNA plasmid (pGenesil1.1-shGrem1) induced GFP expression, indicating successful transfection. Increased expression of GFP was detected 12 h after transfection and peaked at 48 h. At 48 h after transfection, GFP expression was observed through the comparative analysis of bright field images and corresponding fluorescent images and about 60% (Gremlin plasmid) and 65% (Gremlin siRNA plasmid) of the MMCs were found to have been transfected successfully (Figure 2A, 2B). To determine the efficiency of plasmids, Gremlin mRNA and protein levels were assessed using RT-PCR and western blot analysis at 48 h after transfection. Gremlin mRNA was found to be markedly up-regulated to 1.60 ± 0.09 fold (*P* < 0.01, vs NG) in Gremlin plasmid group (Figure 2C) and inhibited to 0.27 ± 0.12 fold (*P* < 0.01, vs NG) in the Gremlin siRNA plasmid group (Figure 2D). We also found that transfection with Gremlin plasmid into MMCs significantly increased the level of Gremlin protein (*P* < 0.01) over that of the NG group (Figure 2E), and transfection with Gremlin siRNA plasmid into MMCs significantly inhibited the level of Gremlin protein (*P* < 0.05) relative to the NG group (Figure 2F). These findings reveal that the plasmids were all effective and suitable for use in the study.

### Effects of Gremlin on cell proliferation and ECM accumulation in MMCs

**Figure 3:**
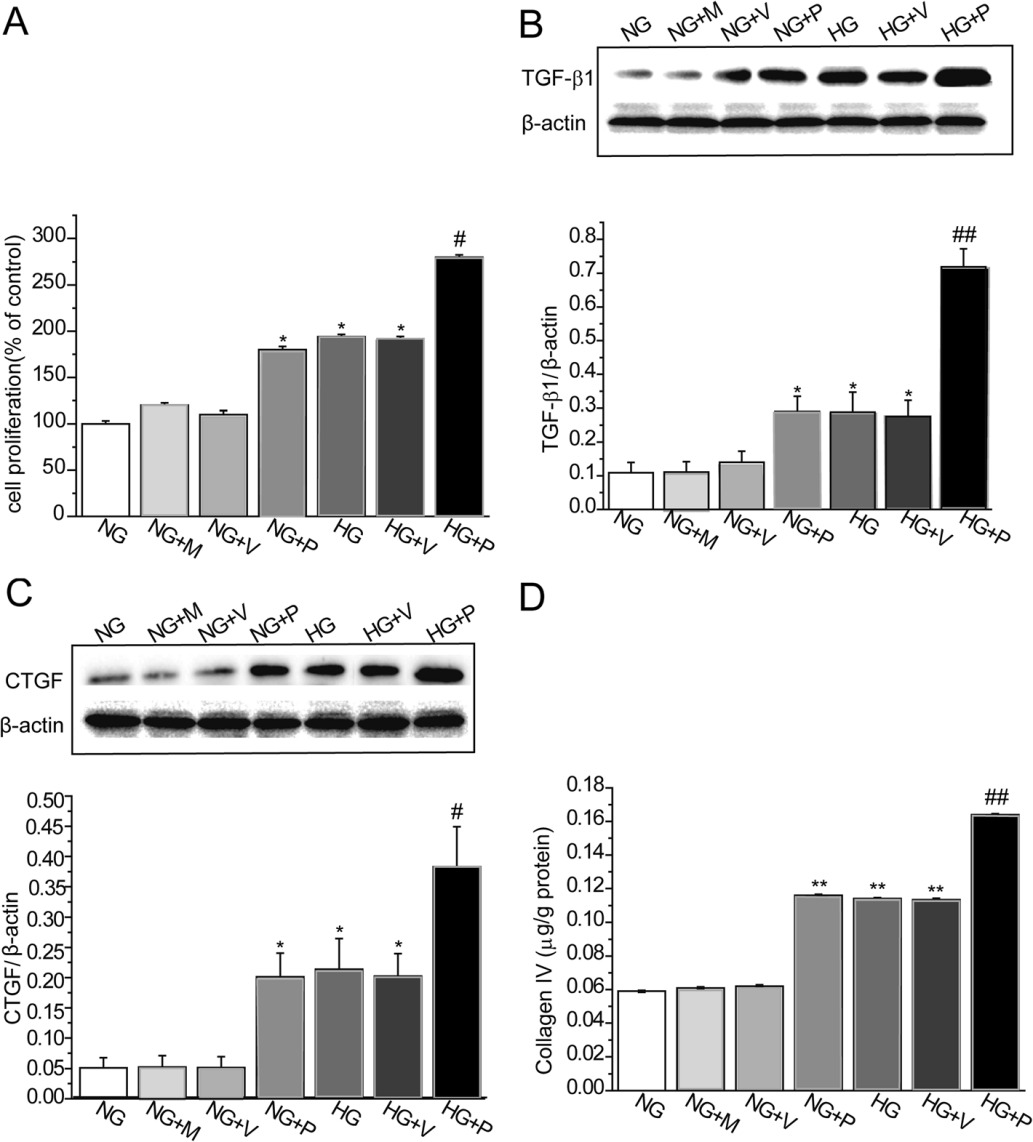
**Effects of Gremlin plasmid on cell proliferation and TGF-β1, CTGF and collagen IV expression in MMCs.** MMCs were transfected with Gremlin (pEGFP-N1-Grem1) or corresponding negative control (pEGFP-N1) plasmids and incubated with HG (30 mM D-glucose) for 24 h. (**A**) Cell proliferation was examined using BrdU ELISA. Total (**B**) TGF-β1 and (**C**) CTGF proteins levels were determined using western blot analysis. (**D**) Culture medium was collected for measurement of collagen IV concentration by radioimmunoassay. NG: MMCs incubated with 5.5 mM D-glucose; NG + M: MMCs incubated with 5.5 mM D-glucose plus 24.5 mM mannitol; NG + V: MMCs transfected with pEGFP-N1 plasmid and incubated with 5.5 mM D-glucose; NG + P: MMCs transfected with pEGFP-N1-Grem1 plasmid and incubated with 5.5 mM D-glucose; HG: MMCs incubated with 30 mM D-glucose; HG + V: MMCs transfected with pEGFP-N1 plasmid and incubated with 30 mM D-glucose; HG + P: MMCs transfected with pEGFP-N1-Grem1 plasmid and incubated with 30 mM D-glucose. Values are expressed as means ± S.E.M. *P < 0.05,**P < 0.01, vs. NG, ^#^*P* < 0.05, ^##^*P* < 0.01, vs. HG.

The effects of Gremlin gene expression on MMC proliferation and ECM accumulation after exposure to high concentrations of glucose where investigated. This was carried out *in vitro* following transfection of MMCs with a plasmid carrying the Gremlin gene. Cell proliferation was determined using BrdU ELISA. Cell proliferation was found to be significantly higher in the NG + P (P < 0.05), HG (P < 0.05) and HG + V (P < 0.05) groups compared with the NG group. Transfection with Gremlin plasmid into MMCs exposed to HG significantly increased HG-induced cell proliferation further (P < 0.05) (Figure 3A). MMCs in the NG + P (P < 0.05), HG (*P* < 0.05) and HG + V (*P* < 0.05) groups showed significantly higher TGF-β1 protein expression levels compared with the NG group. Transfection with Gremlin plasmid into MMCs exposed to HG significantly increased HG-induced overexpression of TGF-β1 protein (*P* < 0.01, vs. HG) (Figure 3B). MMCs in the NG + P (P < 0.05), HG (*P* < 0.05) and HG + V (*P* < 0.05) groups showed significantly higher CTGF protein expression levels compared with the NG group. Transfection with Gremlin plasmid into MMCs exposed to HG significantly increased HG-induced overexpression of CTGF protein (*P* < 0.05, vs. HG) (Figure 3C). Significant accumulation of collagen IV in the culture medium was observed in the NG + P (P < 0.01, vs. NG), HG (*P* < 0.01, vs. NG) and HG + V (*P* < 0.01, vs. NG) groups. Transfection with Gremlin plasmid into MMCs exposed to HG significantly increased HG-induced accumulation of collagen IV (*P* < 0.01, vs. HG) (Figure 3D). These findings indicate that Gremlin can increase MMC proliferation and ECM accumulation after exposure to normal and high levels of glucose.

### Effects of Gremlin inhibition on cell proliferation and ECM accumulation in MMCs

**Figure 4:**
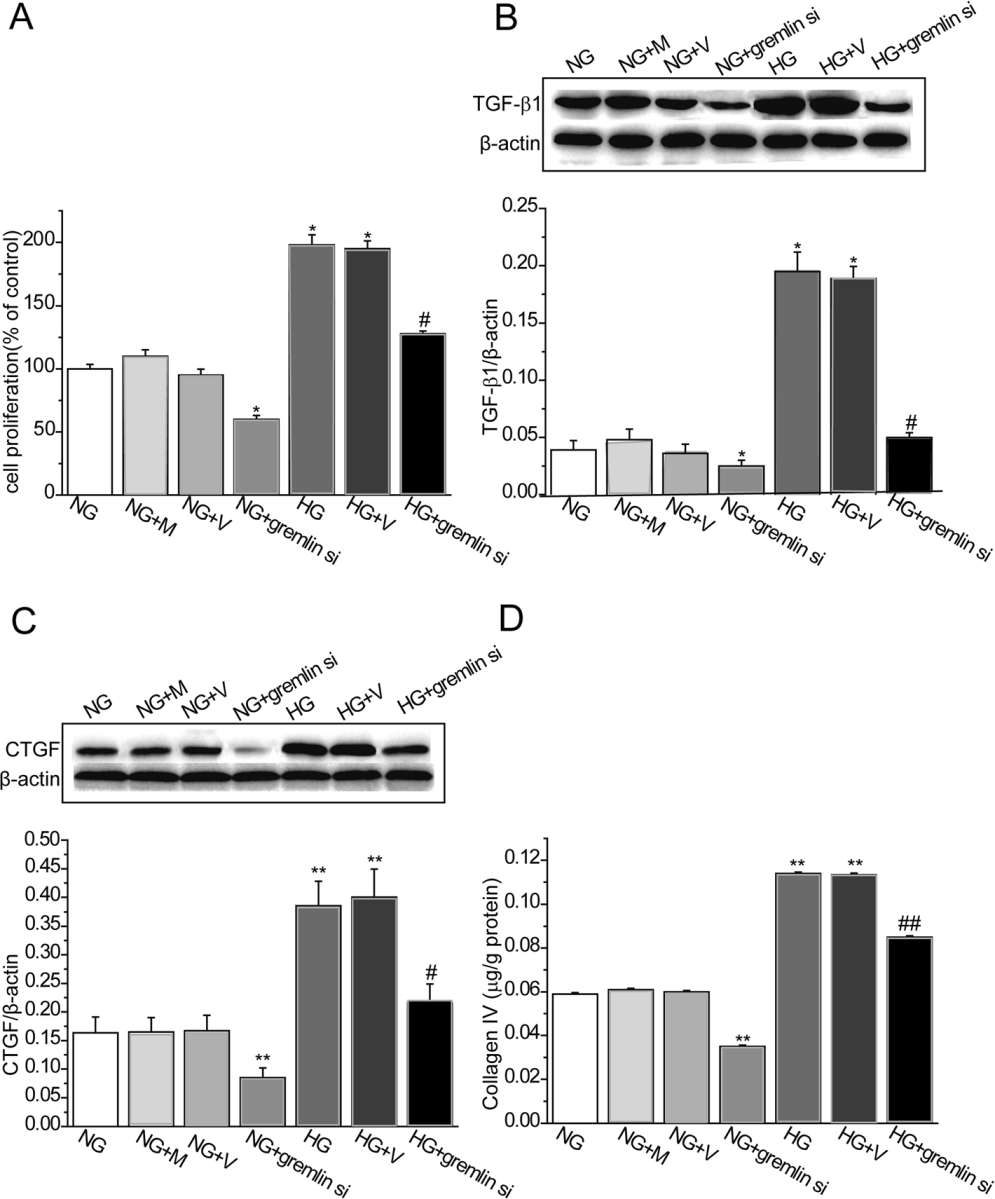
**Effects of Gremlin siRNA plasmid on cell proliferation and TGF-β1, CTGF and collagen IV expression in MMCs.** MMCs were transfected with Gremlin siRNA (pGenesil1.1-shGrem1) or corresponding negative control (pGenesil1.1) plasmids, and incubated with HG (30 mM D-glucose) for 24 h. (**A**) Cell proliferation was examined using BrdU ELISA. Total (**B**) TGF-β1 and (**C**) CTGF protein levels were determined using western blot analysis. (**D**) Culture medium was collected for measurement of collagen IV concentration by radioimmunoassay. NG: MMCs incubated with 5.5 mM D-glucose; NG + M: MMCs incubated with 5.5 mM D-glucose plus 24.5 mM mannitol; NG + V: MMCs transfected with pGenesil1.1 plasmid and incubated with 5.5 mM D-glucose; NG + gremlin si: MMCs transfected with pGenesil1.1-shGrem1 plasmid and incubated with 5.5 mM D-glucose; HG: MMCs incubated with 30 mM D-glucose; HG + V: MMCs transfected with pGenesil1.1 plasmid and incubated with 30 mM D-glucose; HG + gremlin si: MMCs transfected with pGenesil1.1-shGrem1 plasmid and incubated with 30 mM D-glucose. Values are expressed as means ± S.E.M. *P < 0.05, **P < 0.01, vs. NG, ^#^*P* < 0.05, ^##^*P* < 0.01, vs. HG.

Cell proliferation was detected using BrdU ELISA. Transfection of the Gremlin siRNA plasmid into MMCs exposed to NG significantly inhibited cell proliferation (P < 0.05, vs. NG). Cell proliferation was found to be significantly higher in the HG (P < 0.05) and HG + V (P < 0.05) groups compared with the NG group. Transfection of the Gremlin siRNA plasmid into MMCs exposed to HG significantly inhibited HG-induced cell proliferation (P < 0.05, vs. HG) (Figure 4A). Transfection with Gremlin siRNA plasmid into MMCs exposed to NG significantly inhibited expression of TGF-β1 protein (P < 0.05, vs. NG). MMCs in the HG (*P* < 0.05) and HG + V (*P* < 0.05) groups showed significantly higher levels of TGF-β1 protein compared with the NG group. Transfection with Gremlin siRNA plasmid into MMCs exposed to HG significantly inhibited HG-induced overexpression of TGF-β1 protein (*P* < 0.05, vs. HG) (Figure 4B). Transfection with Gremlin siRNA plasmid into MMCs exposed to NG significantly inhibited expression of CTGF protein (P < 0.01, vs. NG). MMCs in the HG (*P* < 0.01) and HG + V (*P* < 0.01) groups showed significantly higher CTGF protein levels compared with the NG group. Transfection with Gremlin siRNA plasmid into MMCs exposed to HG significantly inhibited the HG-induced overexpression of CTGF protein (*P* < 0.05, vs. HG) (Figure 4C). Transfection with Gremlin siRNA plasmid into MMCs exposed to NG significantly inhibited accumulation of collagen IV (P < 0.01, vs. NG). Significant accumulation of collagen IV in the culture medium was observed in the HG (*P* < 0.01, vs. NG) and HG + V (*P* < 0.01, vs. NG) groups. Transfection with Gremlin siRNA plasmid into MMCs exposed to HG significantly reduced the accumulation of collagen IV (*P* < 0.01, vs. HG) (Figure 4D). These findings indicate that inhibition of Gremlin can normalize MMC proliferation and ECM accumulation after exposure to high concentrations of glucose, indicating that Gremlin inhibition may have beneficial effects.

### Effect of Gremlin on activation of the ERK1/2 pathway in MMCs

**Figure 5:**
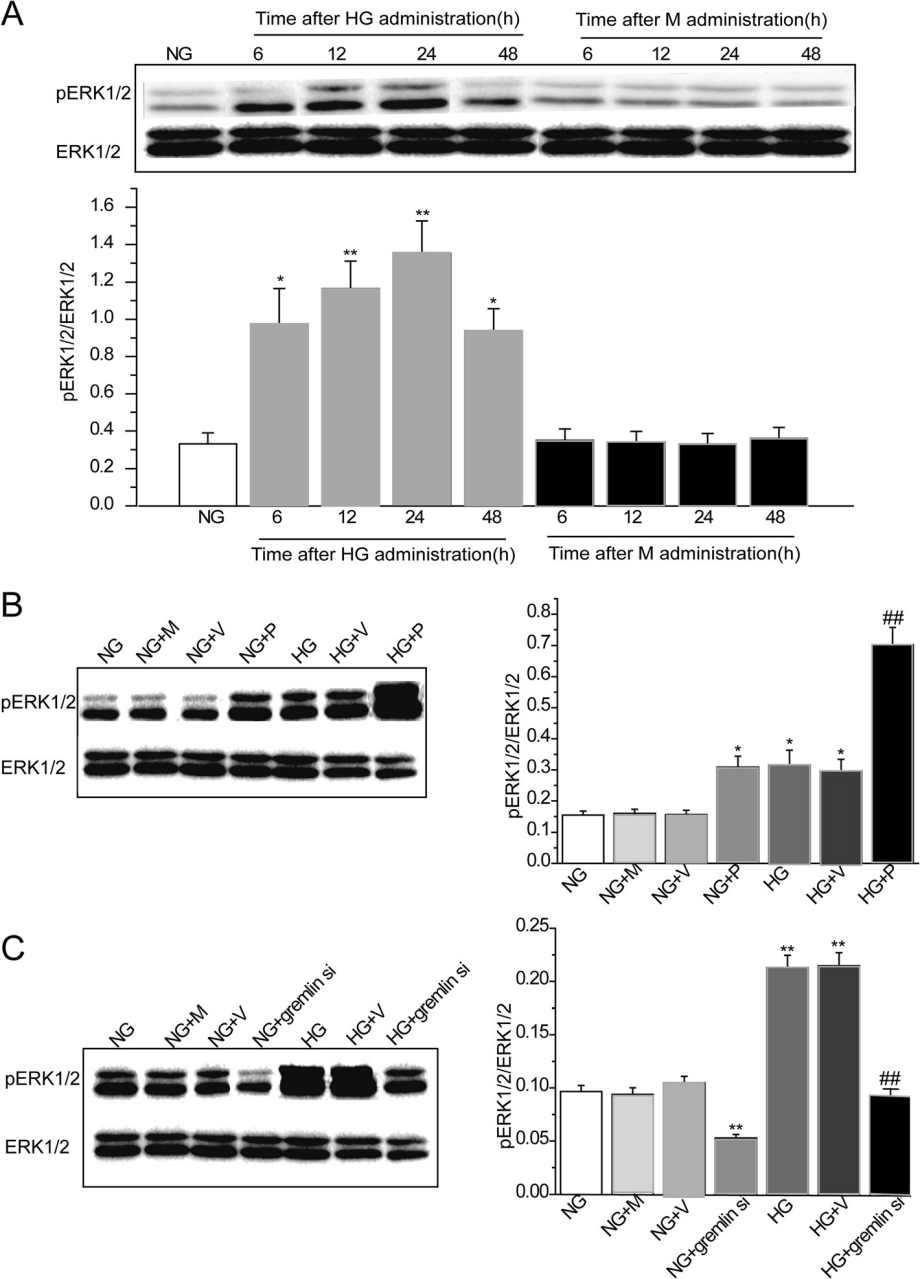
**Effects of Gremlin on pERK1/2 protein expression in MMCs.** MMCs were cultured in NG then stimulated (M) or HG for 6, 12, 24, and 48 h. At the end of each time point, total cell protein contents were extracted for assessment of pERK1/2 protein expression. MMCs were transfected with Gremlin (pEGFP-N1-Grem1), Gremlin siRNA (pGenesil1.1-shGrem1) or their corresponding negative control (pEGFP-N1 or pGenesil1.1, respectively) plasmids, and incubated with HG for 24 h. Protein was then extracted from the cells for evaluation of pERK1/2 protein expression. (**A**) The level of pERK1/2 protein expression was assessed using western blot analysis in MMCs incubated with HG for the indicated times (0–48 h). Values are expressed as means ± S.E.M. *P < 0.05, **P < 0.01 vs. NG. (**B**) MMCs were transfected with Gremlin or the corresponding negative control plasmids and the expression of pERK1/2 protein was assessed by western blot analysis 24 h after HG stimulation. (**C**) MMCs were transfected with Gremlin siRNA or the corresponding negative control plasmids and the expression of pERK1/2 protein was assessed using western blot analysis 24 h after HG stimulation. NG: MMCs incubated with 5.5 mM D-glucose; NG + M: MMCs incubated with 5.5 mM D-glucose plus 24.5 mM mannitol; NG + V: MMCs transfected with pEGFP-N1/pGenesil1.1 plasmids and incubated with 5.5 mM D-glucose; NG + P: MMCs transfected with pEGFP-N1-Grem1 plasmid and incubated with 5.5 mM D-glucose; NG + gremlin si: MMCs transfected with pGenesil1.1-shGrem1 plasmid and incubated with 5.5 mM D-glucose; HG: MMCs incubated with 30 mM D-glucose; HG + V: MMCs transfected with pEGFP-N1/pGenesil1.1 plasmids and incubated with 30 mM D-glucose; HG + P: MMCs transfected with pEGFP-N1-Grem1 plasmid and incubated with 30 mM D-glucose; HG + gremlin si: MMCs transfected with pGenesil1.1-shGrem1 plasmid and incubated with 30 mM D-glucose. Values are expressed as means ± S.E.M. *P < 0.05,**P < 0.01, vs. NG, ^#^*P* < 0.05,^##^*P* < 0.01, vs. HG.

In order to confirm the effect of glucose exposure on ERK1/2 pathway activation in MMCs, cells were cultured in media containing 5.5 mM glucose (NG), prior to stimulation with NG plus 24.5 mM mannitol (M) or 30 mM glucose (HG) for 6, 12, 24 and 48 h. At the end of each time point, pERK1/2 protein levels were evaluated using western blot analysis. We found levels of pERK1/2 protein increased during the first 6 h after HG stimulation (*P* < 0.05, vs. NG), peaking at 24 h (*P* < 0.01, vs. NG) (Figure 5A). In addition, no differences were found in MMCs cultured under conditions of NG plus mannitol among different time points. These findings indicate that HG can induce activation of the ERK1/2 pathway in MMCs. In order to confirm the effect of Gremlin on activation of ERK1/2 pathway in MMCs, we performed *in vitro* analysis, by transfecting Gremlin plasmid and Gremlin siRNA plasmid into MMCs exposed to NG and HG conditions, after which pERK1/2 protein levels were assessed by western blot analysis. MMCs in the NG + P (P < 0.05), HG (*P* < 0.05) and HG + V (*P* < 0.05) groups showed significantly higher protein levels of pERK1/2 compared with the NG group. Transfection with Gremlin plasmid in MMCs exposed to HG significantly induced HG-induced overexpression of pERK1/2 (*P* < 0.01, vs. HG) (Figure 5B). Transfection with Gremlin siRNA plasmid in MMCs exposed to NG significantly inhibited expression of pERK1/2 (P < 0.01 vs. NG). MMCs in the HG (P < 0.01) and HG + V (P < 0.01) groups showed significantly higher protein levels of pERK1/2 compared with the NG group. Transfection with Gremlin siRNA plasmid in MMCs exposed to HG significantly inhibited the HG-induced overexpression of pERK1/2 (*P* < 0.01, vs. HG) (Figure 5C). These findings indicate that Gremlin can induce activation of the ERK1/2 pathway in MMCs.

## Discussion

In the present study, we found that HG up-regulates the expression of Gremlin in MMCs and that Gremlin induces cell proliferation and accumulation of ECM in MMCs. We also provide the first piece of evidence showing that Gremlin enhances the ERK1/2 pathway activation in MMCs.

Increased expression of Gremlin was recently detected in experimental models of DN [7]. Gremlin was first named as *Induced in High Glucose 2* (IHG-2) in mesangial cells exposed to high extracellular glucose *in vitro*. Subsequent *in silico* cloning revealed IHG-2 to be human Gremlin [7,12]. Increased Gremlin expression has also been demonstrated in human mesangial cells exposed to cyclic mechanical strain *in vitro* and in both streptozotocin-induced DN and the 5/6 nephrectomy model of glomerular hypertension *in vivo*[7]. In this study, we show HG time dependently up-regulates mRNA and protein levels of Gremlin in MMCs, suggesting that Gremlin expression is induced by HG.

Recent *in vitro* and *in vivo* evidence suggests that Gremlin participates in DN [13]. Human DN is associated with significantly increased Gremlin expression relative to normal or minimally changed kidneys; Gremlin expression was most obvious in the areas associated with interstitial fibrosis [6]. The co-localization of Gremlin and TGF-β1 expression in glomeruli and tubular cells suggests that Gremlin may be important to mediating some of the pathological effects of TGF-β1 [14]. TGF-β, when added to serum-restricted human mesangial cells, has been found to augment Gremlin expression, but the stimulatory effect of high glucose on Gremlin expression was attenuated by the addition of anti-TGF-β antibody [7]. This suggests that Gremlin is induced by TGF-β under diabetic conditions. Certain Gremlin gene variants are associated with DN, and Gremlin is implicated in the pathogenesis of DN [15]. These data suggest a pathogenetic role for Gremlin in DN and identify Gremlin as a potential therapeutic target. Accumulating amounts of evidence suggest that cell proliferation and ECM synthesis, which are characteristics of mesangial cell activation, occur in DN and cause interstitial fibrosis [16,17]. Early renal hypertrophy, which results partially from cell proliferation, acts as a pacemaker for subsequent irreversible structural changes, such as glomerulosclerosis and tubulointerstitial fibrosis [18]. Second, the fibrotic cytokines TGF-β1 and CTGF are important to the glomerular accumulation of ECM and can induce persistent fibrosis [19-21]. Blockage of these cytokines has shown some promise in human diabetic kidney disease [22]. We successfully constructed a recombinant expression plasmid of Gremlin, pEGFP-N1-Grem1, performed an experiment in which MMCs overexpressed Gremlin RNA, and evaluated its effects on cell proliferation and ECM accumulation under high-glucose conditions. Our results demonstrated that transfection with Gremlin plasmid to MMCs exposed to high levels of glucose increased cell proliferation and induced expression of TGF-β1, CTGF, and collagen IV, indicating that Gremlin plays a significant role in cell proliferation and ECM accumulation.

Gremlin expression is enhanced in mesangial cells cultured under high-glucose conditions, and inhibition of Gremlin exerts beneficial effects on the diabetic kidney. Zhang et al [23] showed that inhibition of Gremlin exerted beneficial effects on the diabetic kidney mainly through maintenance of BMP-7 activity. In a streptozotocin-induced model of type 1 diabetes, knockout mice heterozygous for Grem1 gene deletion (Grem1^+/-^) exhibit protection from the progression of diabetic kidney disease [24]. Inhibition of Gremlin expression has been shown to block TGF-β-induced ECM accumulation [25,26]. RNAi, also known as RNA interference, is a powerful tool for silencing gene expression. Short interfering 21–23-mer double-stranded RNA segments (siRNA) guide mRNA degradation in a sequence-specific fashion [27]. Unlike other types of RNAi, such as RNAi molecular synthesis *in vitro* or the construction of expression frames, shRNA can be used to construct RNAi DNA template expression vectors directly *in vitro*, and it is more stable. shRNA in mammal cells not only blocks the expression of target genes but it is also stably passed on to subsequent generations. It has replaced traditional gene knockout techniques in the establishment of transgenic cell lines and transgenic animals. For these reasons, the construction of shRNA expression vectors has become the most commonly used RNAi technique. We successfully constructed recombinant expression plasmid for Gremlin and pGenesil1.1-shGrem1, performed an experiment involving RNA interference inhibition of the overexpression of Gremlin by transfection of eukaryotic expression vector in MMCs under high-glucose conditions. The results showed that the presence of the Gremlin siRNA plasmid reversed high-glucose induced abnormalities, including increased cell proliferation and induced expression of TGF-β1, CTGF, and collagen IV in MMCs exposed to high levels of glucose, indicating beneficial effects of Gremlin inhibition.

Little is known about the relationship between Gremlin and the activation of the ERK1/2 signal pathway. ERK1/2 is known as an important kinase in the intracellular signal transduction system leading to cell proliferation and protein synthesis [28]. The ERK1/2 pathway is activated in diabetic glomeruli or glomerular mesangial cells cultured under high-glucose conditions and it may contribute to the development of DN [29]. The activation of the ERK1/2 pathway is a requirement for the hyperglycemia-induced production of TGF-β1 and CTGF in MMCs [30]. In this way, the ERK pathway plays a key role in DN. High concentrations of glucose were also associated with increased levels of Gremlin mRNA in bovine retinal pericytes; Gremlin expression was modulated by inhibition of ERK1/2 activation, suggesting that Gremlin may play a role in the regulation of the ERK1/2 pathway [31]. Stabile et al [32] reported that Gremlin up-regulated pERK1/2 expression in subcutaneous microvascular endothelial cells ( SIE ) stimulated by recombinant murine Drm/ Gremlin (rDrm) (50 ng/ml) for 0–2012;60 min. pERK1/2 is a marker of ERK1/2 signal pathway activation. Based upon our data, we concluded that high levels of glucose induced the activation of the ERK1/2 pathway in the MMCs. These are the first findings that show that the transfection with Gremlin plasmid can significantly up-regulate HG-induced overexpression of pERK1/2 in MMCs, and Gremlin siRNA plasmid reversed this abnormality, suggesting that Gremlin induced the activation of the ERK1/2 pathway.

The issue of whether Gremlin has BMP-independent effects on the pathogenesis of DN must be addressed. A number of studies have reported that Gremlin can interact directly with cell surface proteins, such as Slit protein family members or cell-surface heparan-sulfate proteoglycans, to alter cell function, indicating a mechanism of action for Gremlin that is independent of BMP antagonism. Chen et al [33] identified Slit1 and Slit2 as Gremlin-interacting proteins. BMP2 or BMP4 did not interfere with the Gremlin-Slit interaction, suggesting that the region of Gremlin protein responsible for BMP-binding is distinct from that interacting with Slit proteins. In a second study, Stabile et al [32] showed that Gremlin binds with high affinity to the surface of subcutaneous microvascular endothelial cells via uncharacterized cell-surface heparan-sulfate proteoglycans (HS-PGs). Molar excess of BMP4 did not hinder Gremlin-proteoglycan binding, again suggesting that different sites on the Gremlin protein, other than those binding BMPs, are involved. Furthermore, Gremlin proteoglycan binding was shown to cause tyrosine phosphorylation of ERK1/2 in these systemic endothelial cells. HS-PG is the main component of the glomerular capillary basement membrane, mesangial cell and vascular wall, and plays an important role in maintenance of their structural integrity. Our study shows that Gremlin induces ERK1/2 pathway activation in mesangial cells cultured under high glucose exposure, which indicates that Gremlin might interact directly with mesangial cell surface HS-PGs to alter cell function through BMP-independent pathways. Gremlin was also found to bind VEGF receptor-2 (VEGFR2), the main transducer of VEGF-mediated angiogenic signals, in a BMP-independent manner. In this way, Gremlin represents a novel proangiogenic VEGFR2 agonist, distinct from VEGF family ligands. It has implications in vascular development, angiogenesis-dependent diseases and tumor neovascularization [34]. Gremlin was also found to be overexpressed in various human tumors including carcinomas of the cervix, endometrium, lung, ovary, kidney, breast, colon and pancreas [35,36]. It was recently reported that Gremlin induces BMP-independent tumor cell proliferation, migration and invasion [37]. The ability of Gremlin to regulate cell growth via a BMP-independent pathway in DN merits further study.

## Conclusion

In conclusion, we observed that HG up-regulates the expression of Gremlin *in vitro*. Gremlin in turn induces cell proliferation and accumulation of ECM in MMCs. This is the first piece of evidence to show that Gremlin enhances the ERK1/2 pathway activation in MMCs.

## Abbreviations

BMP: Bone morphogenetic protein; MMCs: Mouse mesangial cells; HG: High glucose; DN: Diabetic nephropathy; TGF-β1: Transforming growth factor-β1; CTGF: Connective tissue growth factor; ERK1/2: Extracellular signal-regulated kinase 1/2.

## Competing interests

The authors have no competing interests to declare.

## Authors’ contributions

All of the authors contributed to the writing of the manuscript and approved of the final version. HXH performed the experiments and data analysis, participated in the design of the study and drafted the manuscript. YL and HYH conceived of and designed the study and supervised the work. MDL and YHS contributed to the design of the study, and helped with performing the experiments and drafting the manuscript. YQC and TZ provided intellectual input to the study and helped with the revision of the manuscript. All authors read and approved the final manuscript.

## Pre-publication history

The pre-publication history for this paper can be accessed here:

http://www.biomedcentral.com/1471-2369/14/33/prepub

## References

[B1] Topol LZ Bardot B Zhang Q Resau J Huillard E Marx M Calothy G Blair DG Biosynthesis, post-translation modification, and functional characterization of Drm/Gremlin J Biol Chem 2000 275 12 8785 8793 10.1074/jbc.275.12.8785 10722723

[B2] Martinez G Bertram JF Organisation of bone morphogenetic proteins in renal development Nephron Exp Nephrol 2003 93 1 e18 22 10.1159/000066649 12411745

[B3] Topol LZ Marx M Laugier D Bogdanova NN Boubnov NV Clausen PA Calothy G Blair DG Identification of drm, a novel gene whose expression is suppressed in transformed cells and which can inhibit growth of normal but not transformed cells in culture Mol Cell Biol 1997 17 8 4801 4810 9234736 10.1128/mcb.17.8.4801 PMC232332

[B4] Topol LZ Modi WS Koochekpour S Blair DG DRM/GREMLIN (CKTSF1B1) maps to human chromosome 15 and is highly expressed in adult and fetal brain Cytogenet Cell Genet 2000 89 1–2 79 84 10894942 10.1159/000015568

[B5] Rider CC Mulloy B Bone morphogenetic protein and growth differentiation factor cytokine families and their protein antagonists Biochem J 2010 429 1 1 12 10.1042/BJ20100305 20545624

[B6] Hruska KA Guo G Wozniak M Martin D Miller S Liapis H Loveday K Klahr S Sampath TK Morrissey J Osteogenic protein-1 prevents renal fibrogenesis associated with ureteral obstruction Am J Physiol Renal Physiol 2000 279 1 F130 143 10894795 10.1152/ajprenal.2000.279.1.F130

[B7] McMahon R Murphy M Clarkson M Taal M Mackenzie HS Godson C Martin F Brady HR IHG-2, a mesangial cell gene induced by high glucose, is human gremlin. Regulation by extracellular glucose concentration, cyclic mechanical strain, and transforming growth factor-beta1 J Biol Chem 2000 275 14 9901 9904 10.1074/jbc.275.14.9901 10744662

[B8] Kane R Stevenson L Godson C Stitt AW O’Brien C Gremlin gene expression in bovine retinal pericytes exposed to elevated glucose Br J Ophthalmol 2005 89 12 1638 1642 10.1136/bjo.2005.069591 16299147 PMC1772980

[B9] Seger R Krebs EG The MAPK signaling cascade FASEB J 1995 9 9 726 735 7601337

[B10] Haneda M Araki S Togawa M Sugimoto T Isono M Kikkawa R Mitogen-activated protein kinase cascade is activated in glomeruli of diabetic rats and glomerular mesangial cells cultured under high glucose conditions Diabetes 1997 46 5 847 853 10.2337/diabetes.46.5.847 9133554

[B11] Hao J Zhu L Zhao S Liu S Liu Q Duan H PTEN ameliorates high glucose-induced lipid deposits through regulating SREBP-1/FASN/ACC pathway in renal proximal tubular cells Exp Cell Res 2011 317 11 1629 1639 10.1016/j.yexcr.2011.02.003 21320485

[B12] Murphy M Godson C Cannon S Kato S Mackenzie HS Martin F Brady HR Suppression subtractive hybridization identifies high glucose levels as a stimulus for expression of connective tissue growth factor and other genes in human mesangial cells J Biol Chem 1999 274 9 5830 5834 10.1074/jbc.274.9.5830 10026205

[B13] Walsh DW Roxburgh SA McGettigan P Berthier CC Higgins DG Kretzler M Cohen CD Mezzano S Brazil DP Martin F Co-regulation of Gremlin and Notch signalling in diabetic nephropathy Biochim Biophys Acta 2008 1782 1 10 21 10.1016/j.bbadis.2007.09.005 17980714

[B14] Mezzano S Droguett A Burgos ME Aros C Ardiles L Flores C Carpio D Carvajal G Ruiz-Ortega M Egido J Expression of gremlin, a bone morphogenetic protein antagonist, in glomerular crescents of pauci-immune glomerulonephritis Nephrol Dial Transplant 2007 22 7 1882 1890 10.1093/ndt/gfm145 17403698

[B15] McKnight AJ Patterson CC Pettigrew KA Savage DA Kilner J Murphy M Sadlier D Maxwell AP A GREM1 gene variant associates with diabetic nephropathy J Am Soc Nephrol 2010 21 5 773 781 10.1681/ASN.2009070773 20150533 PMC2865734

[B16] Dorado F Velasco S Esparis-Ogando A Pericacho M Pandiella A Silva J Lopez-Novoa JM Rodriguez-Barbero A The mitogen-activated protein kinase Erk5 mediates human mesangial cell activation Nephrol Dial Transplant 2008 23 11 3403 3411 10.1093/ndt/gfn333 18567890

[B17] Hawkins NJ Wakefield D Charlesworth JA The role of mesangial cells in glomerular pathology Pathology 1990 22 1 24 32 10.3109/00313029009061422 2194156

[B18] Wang S Chen Q Simon TC Strebeck F Chaudhary L Morrissey J Liapis H Klahr S Hruska KA Bone morphogenic protein-7 (BMP-7), a novel therapy for diabetic nephropathy Kidney Int 2003 63 6 2037 2049 10.1046/j.1523-1755.2003.00035.x 12753291

[B19] Kaminski KA Szepietowska B Bonda T Kozuch M Mencel J Malkowski A Sobolewski K Kovalchuk O Chyczewski L Szelachowska M CCN2 protein is an announcing marker for cardiac remodeling following STZ-induced moderate hyperglycemia in mice Pharmacol Rep 2009 61 3 496 503 19605949 10.1016/s1734-1140(09)70092-1

[B20] Wang X Shaw S Amiri F Eaton DC Marrero MB Inhibition of the Jak/STAT signaling pathway prevents the high glucose-induced increase in tgf-beta and fibronectin synthesis in mesangial cells Diabetes 2002 51 12 3505 3509 10.2337/diabetes.51.12.3505 12453907

[B21] Yamabe H Osawa H Kaizuka M Tsunoda S Shirato K Tateyama F Okumura K Platelet-derived growth factor, basic fibroblast growth factor, and interferon gamma increase type IV collagen production in human fetal mesangial cells via a transforming growth factor-beta-dependent mechanism Nephrol Dial Transplant 2000 15 6 872 876 10.1093/ndt/15.6.872 10831644

[B22] Twigg SM Mastering a mediator: blockade of CCN-2 shows early promise in human diabetic kidney disease J Cell Commun Signal 2010 4 4 189 196 10.1007/s12079-010-0102-2 21234125 PMC2995133

[B23] Zhang Q Shi Y Wada J Malakauskas SM Liu M Ren Y Du C Duan H Li Y Zhang Y In vivo delivery of Gremlin siRNA plasmid reveals therapeutic potential against diabetic nephropathy by recovering bone morphogenetic protein-7 PLoS One 2010 5 7 e11709 10.1371/journal.pone.0011709 20661431 PMC2908623

[B24] Roxburgh SA Kattla JJ Curran SP O’Meara YM Pollock CA Goldschmeding R Godson C Martin F Brazil DP Allelic depletion of grem1 attenuates diabetic kidney disease Diabetes 2009 58 7 1641 1650 10.2337/db08-1365 19401426 PMC2699858

[B25] Wang S de Caestecker M Kopp J Mitu G Lapage J Hirschberg R Renal bone morphogenetic protein-7 protects against diabetic nephropathy J Am Soc Nephrol 2006 17 9 2504 2512 10.1681/ASN.2006030278 16899516

[B26] Wang S Hirschberg R BMP7 antagonizes TGF-beta -dependent fibrogenesis in mesangial cells Am J Physiol Renal Physiol 2003 284 5 F1006 1013 12676736 10.1152/ajprenal.00382.2002

[B27] Soutschek J Akinc A Bramlage B Charisse K Constien R Donoghue M Elbashir S Geick A Hadwiger P Harborth J Therapeutic silencing of an endogenous gene by systemic administration of modified siRNAs Nature 2004 432 7014 173 178 10.1038/nature03121 15538359

[B28] Meloche S Pouyssegur J The ERK1/2 mitogen-activated protein kinase pathway as a master regulator of the G1- to S-phase transition Oncogene 2007 26 22 3227 3239 10.1038/sj.onc.1210414 17496918

[B29] Sakai N Wada T Furuichi K Iwata Y Yoshimoto K Kitagawa K Kokubo S Kobayashi M Hara A Yamahana J Involvement of extracellular signal-regulated kinase and p38 in human diabetic nephropathy Am J Kidney Dis 2005 45 1 54 65 10.1053/j.ajkd.2004.08.039 15696444

[B30] Wang L Hu GY Shen H Peng ZZ Ning WB Tao LJ Fluorofenidone inhibits TGF-beta1 induced CTGF via MAPK pathways in mouse mesangial cells Pharmazie 2009 64 10 680 684 19947172

[B31] Leask A Signaling in fibrosis: targeting the TGF beta, endothelin-1 and CCN2 axis in scleroderma Front Biosci (Elite Ed) 2009 1 115 122 19482630 10.2741/E12

[B32] Stabile H Mitola S Moroni E Belleri M Nicoli S Coltrini D Peri F Pessi A Orsatti L Talamo F Bone morphogenic protein antagonist Drm/gremlin is a novel proangiogenic factor Blood 2007 109 5 1834 1840 10.1182/blood-2006-06-032276 17077323

[B33] Chen B Blair DG Plisov S Vasiliev G Perantoni AO Chen Q Athanasiou M Wu JY Oppenheim JJ Yang D Cutting edge: bone morphogenetic protein antagonists Drm/Gremlin and Dan interact with Slits and act as negative regulators of monocyte chemotaxis J Immunol 2004 173 10 5914 5917 15528323 10.4049/jimmunol.173.10.5914

[B34] Mitola S Ravelli C Moroni E Salvi V Leali D Ballmer-Hofer K Zammataro L Presta M Gremlin is a novel agonist of the major proangiogenic receptor VEGFR2 Blood 2010 116 18 3677 3680 10.1182/blood-2010-06-291930 20660291

[B35] Namkoong H Shin SM Kim HK Ha SA Cho GW Hur SY Kim TE Kim JW The bone morphogenetic protein antagonist gremlin 1 is overexpressed in human cancers and interacts with YWHAH protein BMC Cancer 2006 6 74 10.1186/1471-2407-6-74 16545136 PMC1459871

[B36] Sha G Zhang Y Zhang C Wan Y Zhao Z Li C Lang J Elevated levels of gremlin-1 in eutopic endometrium and peripheral serum in patients with endometriosis Fertil Steril 2009 91 2 350 358 10.1016/j.fertnstert.2007.12.007 18314105

[B37] Kim M Yoon S Lee S Ha SA Kim HK Kim JW Chung J Gremlin-1 induces BMP-independent tumor cell proliferation, migration, and invasion PLoS One 2012 7 4 e35100 10.1371/journal.pone.0035100 22514712 PMC3325980

